# Benzylmalonyl-CoA dehydrogenase, an enzyme involved in bacterial auxin degradation

**DOI:** 10.1007/s00203-021-02406-3

**Published:** 2021-05-31

**Authors:** Karola Schühle, Martin Saft, Bastian Vögeli, Tobias J. Erb, Johann Heider

**Affiliations:** 1 Laboratory for Microbial Biochemistry, Philipps University of Marburg, 35043 Marburg, Germany; 2 Max Planck Institute for Terrestrial Microbiology, 35043 Marburg, Germany; 3 LOEWE-Center for Synthetic Microbiology, Marburg, Germany

**Keywords:** Benzylmalonyl-CoA dehydrogenase, Anaerobic indoleacetate degradation, Cinnamoyl-CoA carboxylase/reductase, Flavin, Enzyme kinetics

## Abstract

A novel acyl-CoA dehydrogenase involved in degradation of the auxin indoleacetate by *Aromatoleum aromaticum* was identified as a decarboxylating benzylmalonyl-CoA dehydrogenase (IaaF). It is encoded within the *iaa* operon coding for enzymes of indoleacetate catabolism. Using enzymatically produced benzylmalonyl-CoA, the reaction was characterized as simultaneous oxidation and decarboxylation of benzylmalonyl-CoA to cinnamoyl-CoA and CO_2_. Oxygen served as electron acceptor and was reduced to H_2_O_2_, whereas electron transfer flavoprotein or artificial dyes serving as electron acceptors for other acyl-CoA dehydrogenases were not used. The enzyme is homotetrameric, contains an FAD cofactor and is enantiospecific in benzylmalonyl-CoA turnover. It shows high catalytic efficiency and strong substrate inhibition with benzylmalonyl-CoA, but otherwise accepts only a few medium-chain alkylmalonyl-CoA compounds as alternative substrates with low activities. Its reactivity of oxidizing 2-carboxyacyl-CoA with simultaneous decarboxylation is unprecedented and indicates a modified reaction mechanism for acyl-CoA dehydrogenases, where elimination of the 2-carboxy group replaces proton abstraction from C2.

## Introduction

The C3-dicarboxylic acid malonate and its thioesters malonyl-CoA or malonyl-acyl carrier protein (ACP) are important intermediates in the anabolic metabolism of all bacteria and eukarya. Malonyl-CoA is usually produced from acetyl-CoA in an ATP-dependent reaction by the biotin-containing acetyl-CoA carboxylase complex (Gago et al. 2006). Malonyl-CoA then serves (directly or after transfer to ACP) as building block for the synthesis of fatty acids and many other compounds such as flavonoids, stilbenes, and further secondary metabolites (Foster 2012). In addition, some substituted derivatives of malonyl-CoA (Tohge et al. 2007) are used as building blocks for the biosynthesis of special polyketide metabolites (Chan et al. 2009). Examples are methylmalonyl-CoA for producing erythromycin (Staunton and Wilkinson 1997), ethylmalonyl-CoA for phoslactomycin (Chen et al. 2012) or benzylmalonyl-CoA for splenocin biosynthesis (Chang et al. 2015). These compounds are either produced via biotin-dependent carboxylases similar to acetyl-CoA carboxylase (Erb 2011) or via coenzyme B12-dependent rearrangement reactions of the carbon skeletons of the respective CoA-activated dicarboxylic acids (Weichler et al. 2015). Substituted malonyl-CoA derivatives also occur in other important metabolic pathways, e.g., propionate metabolism via methylmalonyl-CoA, the ethylmalonyl-CoA pathway of acetate assimilation (Erb 2011), or anaerobic degradation pathways of alkanes and indoleacetate, which produce alkylmalonyl-CoA (Wilkes et al. 2002) or (2-aminobenzyl)malonyl-CoA as intermediates (Ebenau-Jehle et al. 2012; Schühle et al. 2016).

**Fig. 1:**
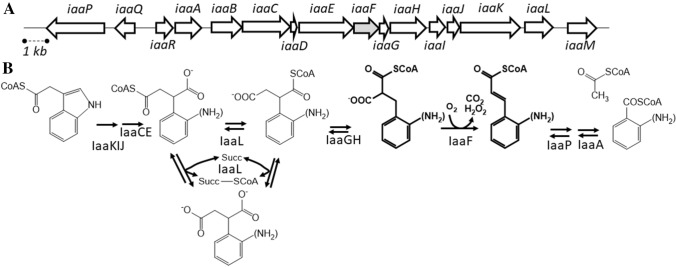
**A** Organisation of the *A. aromatoleum iaa* gene cluster. The genes *iaaQ* and *iaaR* code for transcriptional regulators, *iaaD* for a non-functional truncated protein. **B** Reactions of indoleacetate degradation, starting after uptake (involving the binding protein IaaM) and activation to indoleacetyl-CoA by the CoA ligase IaaB. The reactions of phenylsuccinyl-CoA transferase (IaaL), benzylmalonyl-CoA mutase (IaaGH) and benzylmalonyl-CoA dehydrogenase (IaaF) are shown in detail (IaaF-catalysed reaction highlighted by bold print). Arrows indicate reversible or irreversible steps. Note that in the case of IaaL, both intra- and intermolecular CoA-transfer reactions (with succinate) have been reported (Schühle et al. 2016)

In this study, we investigate benzylmalonyl-CoA dehydrogenase, a member of the acyl-CoA dehydrogenase family, which is encoded by the gene *iaaF* and participates in bacterial indoleacetate degradation (Fig. 1; Ebenau-Jehle et al. 2012; Schühle et al. 2016). Most known acyl-CoA dehydrogenases participate in β-oxidation processes and catalyze the FAD-dependent oxidation of CoA-activated acids to α,β-unsaturated enoyl-CoA intermediates. They usually use an electron-transfer flavoprotein (ETF) as electron acceptor, which transfers the redox equivalents further to ubi- or menaquinones via ETF:quinone oxidoreductases (Ghisla and Thorpe 2004; Frerman 1987). Other members of the family are involved in β-condensation reactions as enoyl-CoA or enoyl-acyl carrier protein (ACP) reductases, using NAD(P)H as reductant (Ghisla and Thorpe 2004), or catalyze apparently unrelated reactions such as desulfination or γ,δ-double bond generation (Schürmann et al. 2015; Blake-Hedges et al. 2020). The function of IaaF in the proposed indoleacetate catabolic pathway is the oxidative decarboxylation of (2-aminobenzyl)malonyl-CoA to 2-aminocinnamoyl-CoA and CO_2_ as shown in Fig. 1. We show in this report that IaaF indeed catalyzes the proposed reaction with benzylmalonyl-CoA, a chemical analogue of the physiological substrate, and propose a catalytic mechanism based on its biochemical properties and sequence comparisons to other acyl-CoA dehydrogenases.

## Materials and methods

### Cloning, heterologous gene expression and preparation of cell-free extracts

The *iaaF* gene (ebA2055) was amplified via PCR from chromosomal DNA of *A. aromaticum* EbN1 using forward and reverse primers (AAGCTCTTCAATGGACTTCGATCTCACCGACG and AAGCTCTTCACCCTGAGCTCACTTCCTTCGCAATG, respectively) and cloned into the vector pAsg-IBA5 (IBA Lifesciences, Göttingen, Germany). The resulting gene codes for a fusion protein of IaaF with a N-terminal Strep-tag. It was produced in *E. coli* DH5α, which was grown in LB medium at room temperature and protein synthesis was induced by anhydrotetracycline addition as reported previously (Schühle et al. 2016). Cells were harvested by centrifugation and resuspended in two volumes of 10 mM Tris/HCl pH 7.5 containing 0.1 mg/ml DNase I. Cell-free extracts were prepared by sonification at 4 °C, followed by ultracentrifugation (100,000 × *g*, 60 min). IaaF activity was present in the soluble fraction, which was used for further purification.

### Protein purification and characterization

Cell-free extracts with overproduced IaaF were applied on a Strep-tag affinity column (IBA Lifesciences, Göttingen, Germany), which was washed with buffer A (25 mM Tris, 150 mM NaCl, pH 7.9) at a flow rate of 1 ml min^−1^ for 5 column volumes, and the enzyme was eluted with buffer A containing 2.5 mM desthiobiotin. The buffer of the purified protein was exchanged into protein storage buffer (30% glycerol, 150 mM NaCl, 25 mM Tris/Cl pH 7.9). Proteins were stored at – 20 °C until further use. Production protocols for the auxiliary enzymes used in this study were previously described as follows: a recombinant Ccr variant from *Caulobacter crescentus* (WP_010920921) carrying three mutations (C146P, I169A, F373G) to open the active site for large substrates (Vögeli et al. 2018); ETF from *A. aromaticum* (CAI09844, CAI09844; Vogt et al. 2019); acyl-CoA oxidase Acx4 from *Arabidopsis thaliana* (NP_160752; Schwander et al. 2016; Vögeli et al. 2018); Etr1P from *Saccharomyces cerevisiae* (Q8WZM3; Rosenthal et al. 2015); and methylmalonyl-CoA epimerase from *Rhodobacter sphaeroides* (ABA79990; Erb et al. 2008). Native molecular masses were determined by applying the proteins to a calibrated gel filtration column (Superdex 200PG, calibration kit HMW, GE Healthcare). Cofactors were extracted from the purified IaaF protein by heat treatment (30 min, 99 °C) and removing the precipitated protein by centrifugation. The supernatant was then analyzed by paper chromatography with Whatman paper (3 mm) using a mobile phase of *n*-butanol:acetic acid:H_2_O (4:1:5) as described in Yagi and Oishi (1971). Retention times were compared with those of standard compounds (FAD, FMN, riboflavin). The quantitation of bound FAD from UV–Vis spectra was as described in Aliverti et al. (1999). Further standard protein analytic techniques, such as SDS–PAGE and protein concentration determinations using Coomassie G-250 dye-binding with bovine serum albumin as standard were performed as described in Coligan et al. (1995).

### Synthesis of benzylmalonyl-CoA and other CoA-thioesters

Benzylmalonyl-CoA was synthesized in a buffer containing 100 mM Tris–HCl pH 8.0, 100 mM KHCO_3_, 20 mM NADPH, 20 µM acyl-CoA oxidase, and 40 μg mL^−1^ carbonic anhydrase, which was supplied with 2.1 μM of the Ccr variant and 1 mM cinnamoyl-CoA (Vögeli et al. 2018). The mixture was incubated for 60 min at 30 °C and then quenched by the addition of 5% (v/v) formic acid. The synthesized benzylmalonyl-CoA was purified by HPLC, using a 1260 Infinity LC system (Agilent) and a Gemini 10 μm NX-C18 AXOA packed column (110 Å, 100 × 21.2 mm, Phenomenex). The protocol used a flow rate of 25 ml*min^−1^ with a basal buffer of 50 mM NH_4_HCO_3,_ pH 8.2 containing 5% methanol for the initial 5 min, followed by a gradual increase from 5 to 40 % MeOH over 15 min, a 2 min washing step at 95% MeOH and re-equilibration of 3 min at 5% MeOH. The purified benzylmalonyl-CoA was lyophilized and stored as powder at – 20 °C until further use. Further substituted malonyl-CoA compounds were generated analogously, as described in Vögeli et al. (2018). Phenylpropionyl-CoA and cinnamoyl-CoA were prepared from the respective acids in two steps: first, phenylpropionic or cinnamic acid was converted to the respective anhydride by solving 200 µmol of the acid and 100 µmol acetic anhydride in 0.5 ml of 100% acetic acid and heating the mixture to 120 °C until the acetic acid was evaporated. Subsequently, the formed anhydrides were converted to CoA-thioesters as described by Schachter and Taggart (1976). The thioesters were further purified by HPLC and lyophilized as described for benzylmalonyl-CoA.

### IaaF enzymatic assays and product analysis

IaaF activity was assayed in 100 mM Tris/HCl buffer at pH 7.8 with added IaaF (5–50 µg/ml) and racemic benzylmalonyl-CoA (0–60 µM). Activity was routinely measured in a continuous photometric assay by directly following the formation of cinnamoyl-CoA at 308 nm (ε = 15.4 mM^−1^ cm^−1^). If indicated, methylmalonyl-CoA epimerase from *Rhodobacter sphaeroides* (Erb et al. 2008; accession number B8XVS7; 0.5 mg/ml) was added after the reaction came to an end. Some experiments also contained artificial electron acceptors such as ferricenium hexafluorophosphate (200 µM), phenazine-methosulfate (100 µM) and dichlorophenyl-indophenol (500 µM), or purified ETF from *A. aromaticum* (1 mg/ml; Vogt et al. 2019) as potential electron acceptors under aerobic or anaerobic conditions. Product formation was confirmed by HPLC analysis after stopping the reactions after defined time periods with added NaHSO_4_ (0.5 M final concentration). The precipitated proteins were removed by centrifugation, and supernatants were applied to a Kinetex 5 µ C18 column (250 × 4.6 mm, Phenomenex). A linear gradient (5–30% acetonitrile in 50 mM NH_4_-formate buffer pH 5.6 over 15 min, flow 0.75 ml/min) was applied, and products were detected by their absorption at 220 and 260 nm. Observed retention times were 7.7 min for benzylmalonyl-CoA, 15.9 min for phenylpropionyl-CoA and 14.2 min for cinnamoyl-CoA. Formation of H_2_O_2_ was quantified using the fluorescence-based Ampliflu Red “easy to use” kit (Sigma–Aldrich; excitation 571 nm, emission 630 nm) according to the user manual.

An alternative coupled photometric assay for IaaF activity was coupled to NADH reduction in the presence of the unspecific enoyl-CoA reductase Etr1P (Rosenthal et al. 2015), which catalyzes the NADH-dependent reduction of a broad variety of enoyl thioesters to acyl thioesters. The assay mixture contained 0.1 mg/ml IaaF, 0.6 mg/ml Etr1P and 0.4 mM NADH and was started by addition of 50–100 µM of various substituted malonyl-CoA-thioesters. Oxidation of NADH was followed at 340 nm (ε = 6.22 mM^−1^ cm^−1^).

Reduction of cinnamoyl-CoA by IaaF (reverse reaction) was tested under anaerobic conditions using 100 mM MOPS/KOH buffer pH 6.8 (for non-carboxylating conditions) or 100 mM bicarbonate/HCl buffer pH 6.8 (for carboxylating conditions). The assays contained 5–25 µg IaaF and 1 mM benzyl- or methyl viologen, which had been fully reduced by adding portions of Na-dithionite from a 10 mM stock solution. Reactions were started by addition of cinnamoyl-CoA (0.5 mM) and oxidation of the reduced viologens was followed photometrically at 600 nm. After reaction times of > 10 min, the assays were also tested for product formation by HPLC analysis.

The rate of spontaneous epimerization of benzylmalonyl-CoA was approximated by determining the rate of hydrogen–deuterium exchange in D_2_O. To this end, 100 µM lyophilized benzylmalonyl-CoA was dissolved in 100 mM Tris-DCl buffer in D_2_O (pD 8.0) and incubated at 15 °C in an HPLC–MS autosampler. At defined time points, samples were analyzed via HPLC–MS (1260 Infinity LC system from Agilent using a Gemini 10 mm NX-C18 110 A˚, 100 × 21.2 mm, AXOA packed column from Phenomenex) and the relative amount of deuterium incorporation into the α-position of benzylmalonyl-CoA was determined from the isotopomer ratios. Even if the benzylmalonyl-CoA used was already a racemic mixture, the determined deuteration rate is expected to reflect the approximate racemization rate, because both processes depend on deprotonation of Cα of benzylmalonyl-CoA.

### Sequence alignment

Amino acid sequences of IaaF orthologues from bacteria and archaea and of further members of the acyl-CoA dehydrogenase family were aligned using Clustal Omega (www.ebi.ac.uk/Tools/msa/clustalo and avermitilis.ls.kitasato-u.ac.jp/clustalo) and bootstrap values were calculated in 1000 replications. A neighbour-joining tree was constructed based on the alignment, using the Program iTOL (itol.embl.de/).

## Results

### Enzymatic synthesis of benzylmalonyl-CoA

Since no CoA ligases or CoA-transferases are available to activate benzylmalonate to the CoA-thioester, we assayed several variants of the Ecr enzyme family for synthesizing benzylmalonyl-CoA via reductive carboxylation of cinnamoyl-CoA (Peter et al. 2015). The prototype enzyme of this family is crotonyl-CoA carboxylase/reductase (Ccr), which catalyzes the reductive carboxylation of crotonyl-CoA to ethylmalonyl-CoA in the recently discovered ethylmalonyl-CoA cycle of acetyl-CoA assimilation (Erb et al. 2007; Erb 2011), but these enzymes also convert CoA-thioesters of various other α,β-unsaturated acids to the corresponding saturated 2-carboxyacyl thioesters (Peter et al. 2015), including cinnamoyl-CoA to benzylmalonyl-CoA (Peter et al. 2016). We obtained the best yields of benzylmalonyl-CoA with a previously reported Ccr from *Caulobacter crescentus* containing three amino acid exchanges and, therefore, used this enzyme to produce the substrate for benzylmalonyl-CoA dehydrogenase (Vögeli et al. 2018; Schwander et al. 2016).

### Stereochemistry of benzylmalonyl-CoA

**Fig. 2:**
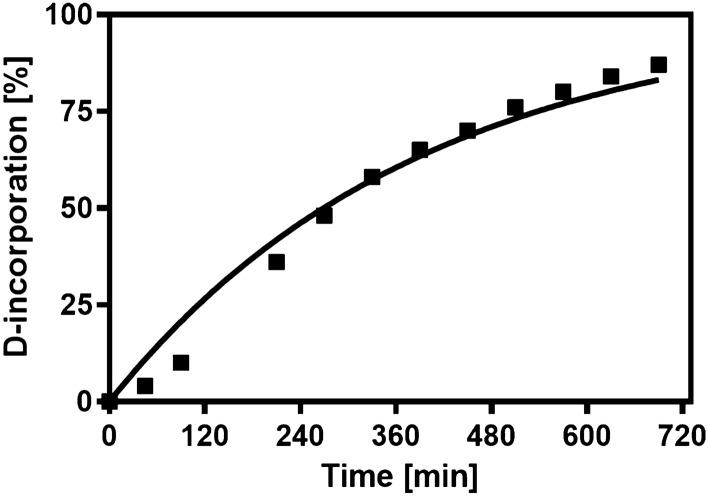
Spontaneous deuteration of enzymatically synthesized benzylmalonyl-CoA. Time-dependent incorporation of deuterium into benzylmalonyl-CoA in D_2_O-based buffer is shown. Curve fitting indicates a half life time of 4.5 h and a rate constant of 4.2 × 10^−5^ s^−1^

Based on the conserved active site geometry of Ccr, we expected that the enzyme generated stereospecifically (*S*)- benzylmalonyl-CoA, yet the obtained product appeared to be a racemic mixture when applied in our further experiments (see below). Therefore, we determined whether benzylmalonyl-CoA spontaneously racemizes in solution by determining the time-dependent exchange of the C-2 proton in D_2_O-based Tris–Cl buffer (pD 8.0) as proxy for the analogous racemization reaction. A gradual increase of deuterium content in benzylmalonyl-CoA from 0 to almost 100% was observed within 13 h at room temperature, indicating a spontaneous deuteration rate of 4.2 × 10^−5^ s^−1^ (Fig. 2), assuming first-order kinetics. Racemization does not involve kinetic isotope effects and, therefore, should occur even faster than deuteration. Because of the time needed for extraction and preparation of benzylmalonyl-CoA, we expect that our experiments were always performed with racemic mixtures, regardless of the enantiomer specificity of Ccr. Unfortunately, the lack of standards prohibited us from identifying which enantiomer was produced by the enzymes.

### Identification of IaaF as a benzylmalonyl-CoA oxidase

**Fig. 3:**
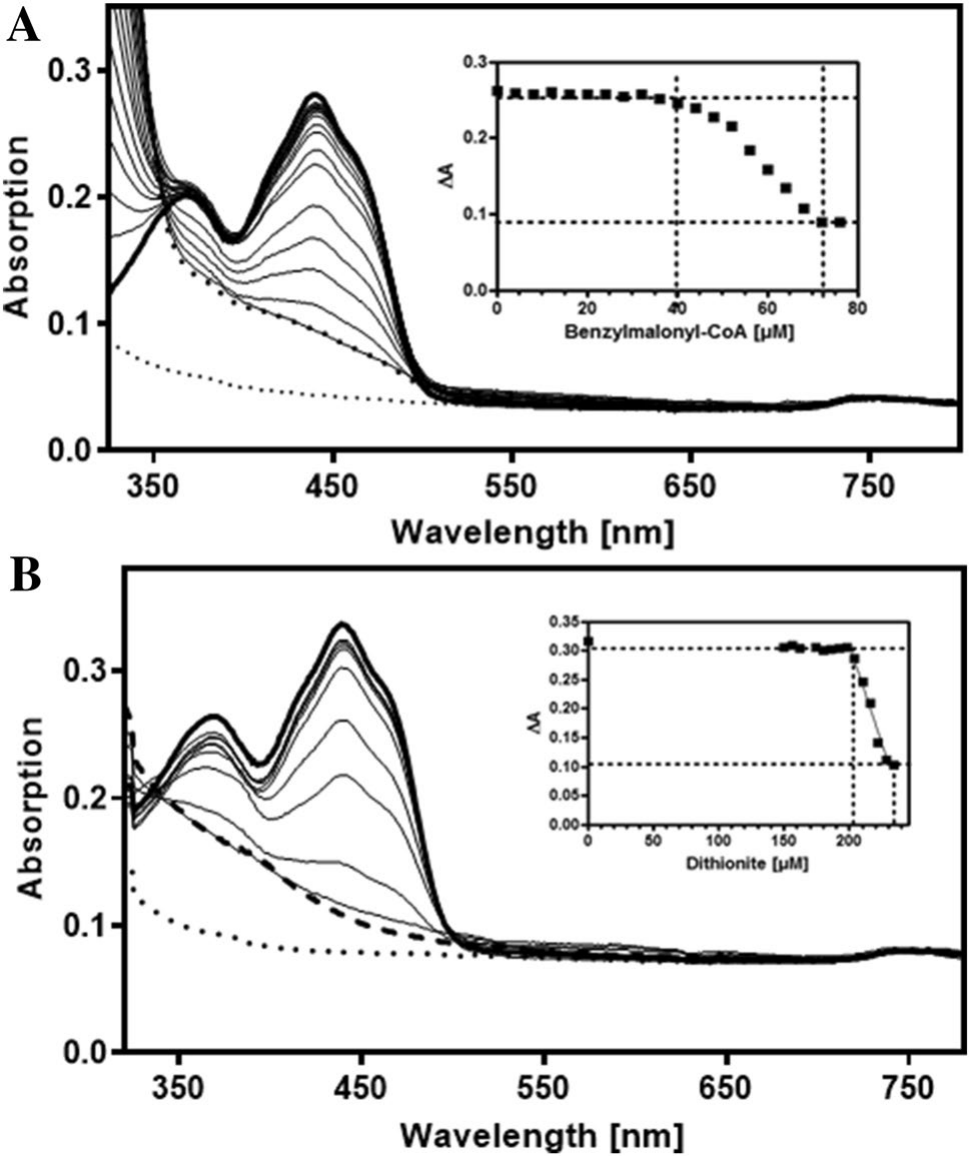
Absorption spectra of IaaF. IaaF concentrations were at 4.5 µM (per holoenzyme), and the tests were done in the absence of methylmalonyl-CoA epimerase. The enzyme was titrated with **A** benzylmalonyl-CoA or **B** dithionite, until fully quenched. Thicker lines indicate the initial (topmost) and final spectra (lowermost, broken line), as well as control spectra of benzylmalonyl-CoA without added enzyme (dotted lines). Note that the strong absorption increase at < 350 nm in **A** represents the increasing production of cinnamoyl-CoA. Inserts indicate the absorption decrease at 450 nm with added concentrations of the reductants

The *iaaF* gene from *Aromatoleum aromaticum* was cloned into vector pAsg5 with a 5’ strep-tag fusion (IBA Lifesciences, Göttingen, Germany), expressed in *E. coli*, and the produced protein was purified by affinity chromatography. We obtained a yellow protein which consisted of a single subunit of the expected size (41 kDa after SDS–PAGE). The native mass of the enzyme was determined as 159 kDa by gel filtration chromatography, suggesting a homotetrameric quaternary structure. Spectrophotometric characterization of the purified protein confirmed the presence of a flavin cofactor, which was extracted by acid-precipitation of the protein and identified by comigration with an FAD reference via paper chromatography. The FAD content was calculated as 4.2 per homotetramer, assuming a molar extinction coefficient of 11.3 mM^−1^ cm^−1^ at the absorption maximum of 450 nm (Leutwein and Heider 2002). The cofactor was fully reduced by stepwise addition of either benzylmalonyl-CoA or dithionite as reductants (Fig. 3). Changes of the spectra occurred only after about 50 µM of benzylmalonyl-CoA or 200 µM of dithionite had been added (Fig. 3), suggesting that dissolved oxygen in the buffer initially acted as electron acceptor, before reduction of the enzyme took effect. After adjusting for this effect, the calculated molar ratios between added reductant and IaaF reduction revealed stoichiometries of 1.2 dithionite and 1.9 benzylmalonyl-CoA needed to reduce one IaaF monomer, respectively (Fig. 3). Although both reductants are two-electron donors, only the dithionite showed the expected stoichiometry for full reduction of the FAD, whereas the value for benzylmalonyl-CoA is about twice as large. This indicates that IaaF reacts stereospecifically with only one enantiomer of the racemic benzylmalonyl-CoA obtained after synthesis and storage (see above). Although the non-reactive enantiomer racemizes spontaneously (see above), the observed rate of deuterium exchange is 1,700-fold lower than the maximum k_cat_ value of IaaF and, therefore, racemization does not take effect within the time-frame of the experiment.

### Catalytic properties of benzylmalonyl-CoA dehydrogenase

Activity of IaaF was assayed photometrically essentially as previously described for benzylsuccinyl-CoA dehydrogenase (Leutwein and Heider 2002). The assays were started with enzymatically produced benzylmalonyl-CoA (see above) with O_2_ serving as electron acceptor, and were evaluated by recording the absorption increase at 308 nm due to the production of cinnamoyl-CoA (using an experimentally determined ε_308_ = 15.4 mM^−1^ cm^−1^). The expected physiological substrate of IaaF during indoleacetate degradation is (2-aminobenzyl)malonyl-CoA, which was not available for testing. However, the missing amino group in benzylmalonyl-CoA does not appear crucial for substrate recognition, since the compound was readily accepted by IaaF and yielded a maximum turnover rate of 2.7 U mg^−1^. We only observed activity in the presence of oxygen and did not observe benzylmalonyl-CoA oxidation coupled to the reduction of typical artificial electron acceptors for acyl-CoA dehydrogenases, such as the ferricenium cation or phenazine-methosulfate/dichlorophenyl-indophenol under aerobic or anaerobic assay conditions. Moreover, IaaF did not react with purified recombinant electron transfer flavoprotein (ETF) from *A. aromaticum*, which serves as physiological electron acceptor for other acyl-CoA dehydrogenases, as recently described by Vogt et al. (2019). While oxygen may be used as electron acceptor for aerobic growth on indoleacetate, it is not clear yet what replaces ETF under denitrifying growth conditions. In contrast to other known acyl-oxidases, e.g., from rat peroxisomes or from *Arabidopsis* (accession numbers 1IS2, 2IX5), IaaF contains a sequence motif very close to the characterized ETF-interaction site of human medium chain acyl-CoA dehydrogenase (Toogood et al. 2004). However, such a motif is also present in sulfinopropionyl-CoA desulfinase (Schürmann et al. 2015), a member of the enzyme family not even catalysing a redox reaction.

**Fig. 4:**
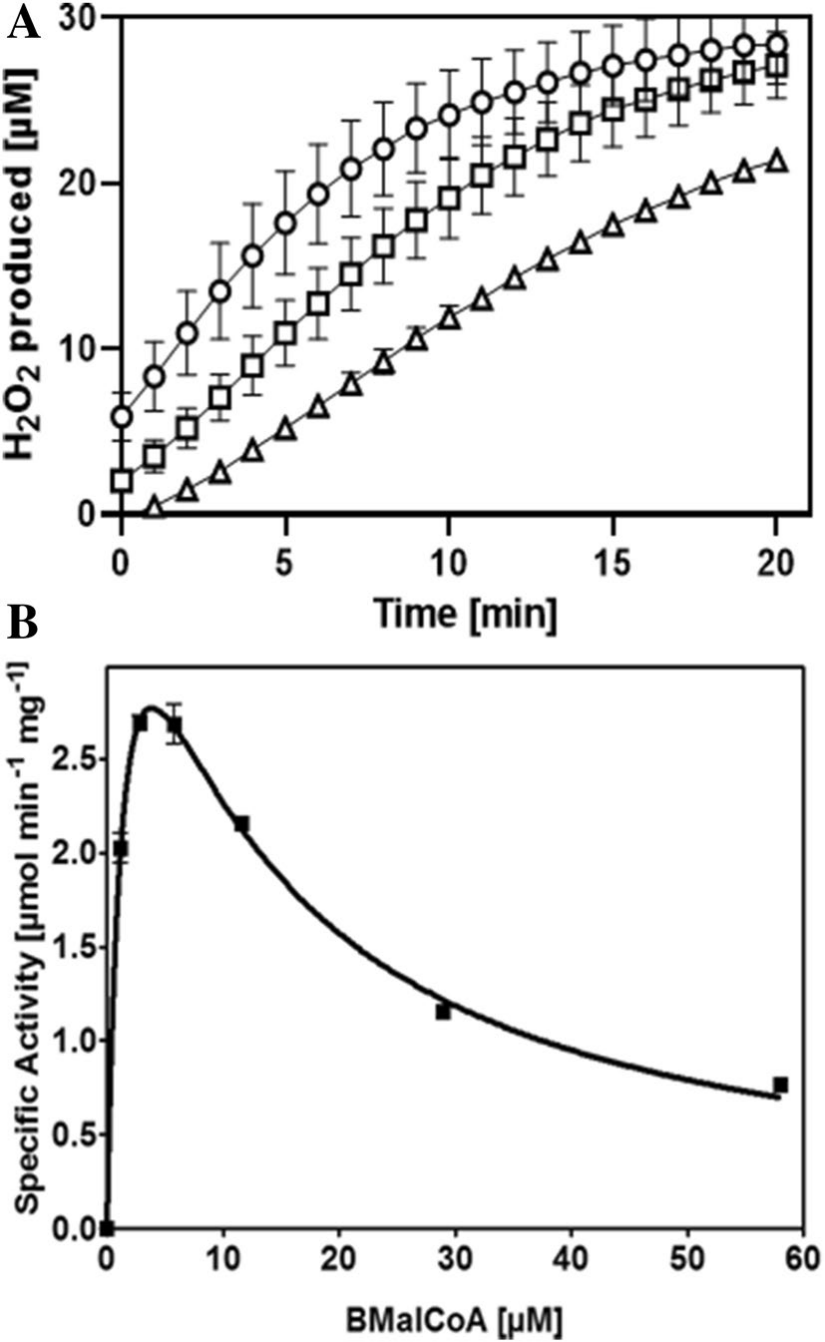
H_2_O_2_ production by IaaF and steady state enzyme kinetics of benzylmalonyl-CoA (BMalCoA) oxidation. **A** Time-dependent H_2_O_2_ production after starting the reaction with 20 µM benzylmalonyl-CoA and different IaaF concentrations (triangles, squares and circles represent 18, 36 and 72 nM IaaF, respectively). **B** Steady state kinetics of IaaF. Standard deviations are indicated by error bars

The photometric assay was confirmed by following the turnover of benzylmalonyl-CoA by HPLC analysis. After starting the reaction by adding the substrate, we observed the decrease of its concentration and the formation of a new CoA-thioester over time which was identified as cinnamoyl-CoA by its UV–Vis spectrum (Peter et al. 2016; Johns 1974). Because *A. aromaticum* grows almost equally well on indoleacetate under aerobic or anaerobic conditions, IaaF may act as an oxidase if oxygen is available. However, it is obvious that it also needs another physiological electron acceptor, which remains unknown. Because of its demonstrated activity with benzylmalonyl-CoA, we propose benzylmalonyl-CoA dehydrogenase as enzyme name, referring to its affiliation to the acyl-CoA dehydrogenase family, which also includes the acyl-CoA oxidases (Kim and Miura 2004). After the reaction with benzylmalonyl-CoA ended, it could be restarted by adding methylmalonyl-CoA epimerase to the assay. This confirms that IaaF is strictly stereospecific and initially converts only one of the benzylmalonyl-CoA enantiomers, while the other one is used only after it is enzymatically epimerized. To our knowledge, this is the first time benzylmalonyl-CoA is reported to be converted by a methylmalonyl-CoA epimerase. As noted before, the non-enzymatic racemization rate of benzylmalonyl-CoA is too slow to interfere in this experiment. None of our experiments produced any trace of phenylpropionyl-CoA, indicating a strict coupling of decarboxylation and oxidation of benzylmalonyl-CoA in the IaaF reaction. The use of oxygen as electron acceptor for benzylmalonyl-CoA oxidation by IaaF suggested the production of H_2_O_2_ in the enzyme assay. Using a fluorescence-based detection system, we indeed confirmed the release of H_2_O_2_ by IaaF in approximately equimolar ratio with the benzylmalonyl-CoA oxidized (Fig. 4A).

Apparent kinetic parameters of IaaF were determined for conversion of benzylmalonyl-CoA to cinnamoyl-CoA, using freshly prepared substrate to prevent significant hydrolysis to benzylmalonate. The data fitted well to a strongly substrate-inhibited Michaelis–Menten enzyme kinetics with a rather low apparent *K*_m_ value of 1.6 ± 0.3 µM for benzylmalonyl-CoA (racemic mixture), an apparent *V*_max_ value of 5.1 ± 0.4 U/mg (equivalent to an apparent k_cat_ of 3.5 s^−1^), and a low apparent substrate inhibition parameter *K*_is_ of 9.1 ± 1.3 µM. Because of the low *K*_is_ value, the enzyme falls short of its theoretical maximum rate, exhibiting a maximal observed rate of only 2.7 U/mg at 7 µM benzylmalonyl-CoA (Fig. 4B). Regarding the racemic nature of the substrate, we cannot discriminate which of the enantiomers is responsible for the observed substrate inhibition.

IaaF was also tested for its reverse activity, i.e., reduction of cinnamoyl-CoA under anaerobic conditions in the presence or absence of CO_2_ (supplied from a 100 mM bicarbonate/CO_2_ buffer at pH 6.8). Dithionite-reduced benzyl or methyl viologen have previously been used as low-potential reductants to provide exergonic redox conditions for driving the reverse reaction of benzylsuccinyl-CoA dehydrogenase (Vogt et al. 2019), but benzylmalonyl-CoA dehydrogenase did not convert cinnamoyl-CoA to either benzylmalonyl-CoA (with carboxylation) or phenylpropionyl-CoA (without carboxylation) under these conditions, as determined by HPLC analysis.

Finally, we performed assays to determine the substrate spectrum of IaaF. To accomplish this, we synthesized various alkyl- or aryl-substituted malonyl-CoA derivatives using several previously described ECR family variants (Vögeli et al. 2018). These compounds were isolated, lyophilized and used as substrates. Experiments were performed as coupled photometric enzyme assays with the enoyl-CoA reductase Etr1p which unspecifically reduces all unsaturated decarboxylation products produced by IaaF with NADH as electron donor (Rosenthal et al. 2015). Activities were measured by recording the absorption decrease at 340 nm due to NADH oxidation, after the assays were started by adding the respective malonyl-CoA derivatives at concentrations of 50 and 100 µM. These concentrations are high enough to cause already strong substrate inhibition in the case of benzylmalonyl-CoA (Fig. 4B) and represent a compromise between providing still measurable IaaF activities, both in controls with benzylmalonyl-CoA and in assays with sterically restrictive alternative substrates with high expected *K*_m_ values. The results are shown in Table 1, indicating that IaaF still showed by far the highest activity with benzylmalonyl-CoA, even under substrate-inhibited conditions.

**Table 1:** Substrate range of IaaF

Substrate concentration	50 µM	100 µM
Benzylmalonyl-CoA	202	144
Hexylmalonyl-CoA	14	18
(3-Methyl)butylmalonyl-CoA	26	41
Butylmalonyl-CoA	19	27
Ethylmalonyl-CoA	nd	nd
Methylmalonyl-CoA	nd	nd
Phenylpropionyl-CoA	nd	nd

The values indicate specific activities with various CoA-thioesters in mU/mg

Note that the assay differs from the standard assay used for benzylmalonyl-CoA conversion, resulting in lower activity values. Standard deviations were lower than 20% of the indicated values

*nd *not detectable

The observed 30% decrease in benzylmalonyl-CoA turnover rates between the assays with 50 and 100 µM substrate is consistent with the recorded substrate inhibition kinetics (Fig. 4B). Using the alternative substrates, we detected low activities of IaaF with hexylmalonyl-CoA, (3-methyl)butylmalonyl-CoA, and butylmalonyl-CoA, whereas no activity was observed with ethylmalonyl-CoA, methylmalonyl-CoA, or phenylpropionyl-CoA. Therefore, IaaF seems to accept several aliphatic alkylmalonyl-CoA analogues with a chain length of four or more C-atoms in straight or branched alkyl chains, but to reject analogues with side chains of only one or two C-atoms. Moreover, it appears crucial that the substrate carries an α-carboxy group, as apparent by the complete inactivity of IaaF with phenylpropionyl-CoA. All alternative substrates show higher turnover rates at 100 µM than at 50 µM concentration, suggesting very high apparent *K*_m_ values and no substrate inhibition effects at the applied concentrations. Because all measured activities with these substrates were rather low and did not appear to have physiological impact, we did not continue with a full enzyme kinetic characterization.

## Discussion

In this manuscript we report on benzylmalonyl-CoA dehydrogenase (IaaF), a new member of the acyl-CoA dehydrogenase family, which catalyses the irreversible oxidative decarboxylation of benzylmalonyl-CoA to cinnamoyl-CoA. The enzyme is involved in the degradation of indoleacetate in *A. aromaticum* and several other bacteria or archaea and is encoded in a common operon with other enzymes of the proposed degradation pathway (Fig. 1; Ebenau-Jehle et al. 2012, Schühle et al. 2016). The enzyme has been proposed to catalyze a coupled oxidation and decarboxylation reaction of (2-aminobenzyl)malonyl-CoA, producing 2-aminocinnamoyl-CoA for further degradation via β-oxidation (Fig. 1). We show here that the enzyme indeed performs the previously proposed reaction, using benzylmalonyl-CoA as proxy for the unavailable physiological substrate. Although the enzyme participates in an anaerobic degradation pathway, the only accepted electron acceptor we could identify is oxygen, which is reduced to H_2_O_2_. Since IaaF accepts neither ETF nor artificial redox dyes such as the ferricenium ion or phenazine-methosulfate as electron acceptors, the re-oxidation mechanism for IaaF still remains unknown. The redox potentials of NAD or NADP are too low to allow direct electron transfer, and a hypothetical electron confurcation mechanism would be dependent of a bifurcation-competent ETF, while the genome of *A. aromaticum* only codes for a single non-bifurcating ETF (Rabus et al. 2005), which is not reactive with IaaF. Remarkably, the predicted *iaa* operons of several strictly anaerobic bacteria like *Desulfatiglans anilini*, other sulfate reducing bacteria or the archaeon *Ferroglobus placidus* contain genes coding for a non-bifurcating ETF paralogue in addition to an *iaaF* orthologue, as previously reported (Schühle et al. 2016). It may be speculated that the *iaa* genes originated in sulfate-reducing bacteria together with a dedicated ETF as electron acceptor for IaaF and were laterally transferred to denitrifyers. These new hosts still use IaaF, but their ETF:quinone oxidoreductases (Frerman 1987) are fundamentally different from those of the strictly anaerobic species (Vogt et al. 2019), and, therefore, a new mechanism for IaaF re-oxidation was needed. We have to assume from our anaerobic growth experiments, that alternative oxidants can be used, but their nature remains unknown for now.

**Fig. 5:**
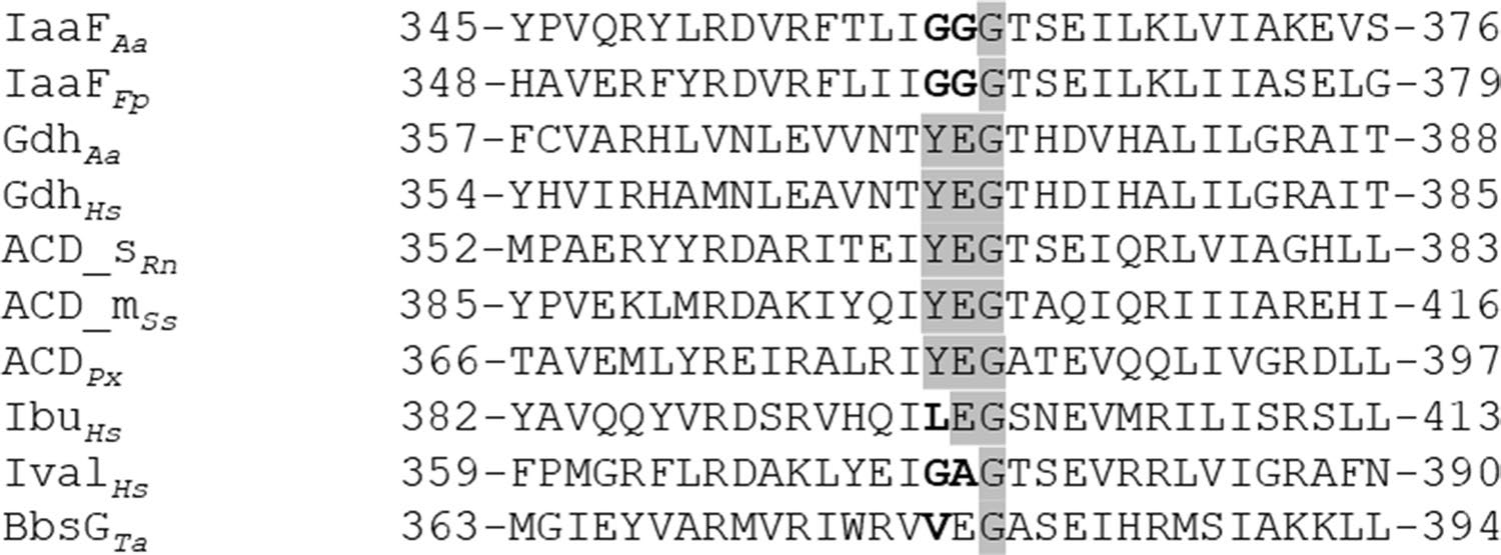
Alignment of the conserved YEG motifs of acyl-CoA dehydrogenases with IaaF orthologues and other deviating members of the enzyme family (deviations highlighted in bold print). IaaF_*Aa*_ and IaaF_*Fp*_, IaaF orthologues of *A. aromaticum* and *Ferroglobus placidus*, respectively; Gdh_*Aa*_ and Gdh_*Hs*_, glutaryl-CoA dehydrogenase from *A. aromaticum* and human, respectively; ACD_s_rat_, ACD_m_pig_ and ACD_*Px*_ acyl-CoA dehydrogenases from rat (short chain), pig (medium chain), and *Paraburkholderia xenovorans*, respectively; Ibu_*Hs*_ and Ival_*Hs*_, human isobutyryl- and isovaleryl-CoA dehydrogenases, respectively; BbsG_*Ta*_, (*E*)-benzylidenesuccinyl-CoA dehydrogenase from *Thauera aromatica*. Numbers indicate the positions in the amino acid sequences. Accession numbers: WP_011236983, WP_012966786, CAI07810, 1SIR_A, 1JQI_A, NP_999204, 5JSC_A, NP_055199, 1IVH_A, AAF89842

The substrate specificity of IaaF appears to be quite narrow. The observed activities with benzylmalonyl-CoA and several aliphatic alkylmalonyl-CoA analogues with a minimum alkyl chain length of four C-atoms indicate that IaaF is able to convert malonyl-CoA derivatives with substituents of similar sizes as an aromatic ring. Therefore, we expect that it also efficiently turns over substituted variants of benzylmalonyl-CoA, such as the physiologically occurring (2-aminobenzyl)malonyl-CoA, although these compounds were not available for testing. The enzyme showed high activity and a very low apparent *K*_m_ value with benzylmalonyl-CoA, as well as severe substrate inhibition at low substrate concentrations. It still needs to be seen whether this inhibition is a physiological effect or is exerted by the “wrong” enantiomer present in the racemic mixture. We have previously also reported substrate inhibition for another enzyme of the pathway, indoleacetate-CoA ligase IaaB (Schühle et al. 2016), and propose that the inhibition properties of these two enzymes may prevent the production of too much CoA-activated benzoate analogues, which may overwhelm the further metabolic reactions, e.g., the rather slow reduction of the aromatic ring via benzoyl-CoA reductase (Fuchs et al. 2011). If (2-aminobenzyl)malonyl-CoA accumulates because of a stalled benzoyl-CoA metabolism, it is expected to be converted backwards to (2-aminophenyl)succinyl-CoA via the two preceding reversible reactions of the pathway (phenylsuccinyl-CoA mutase IaaGH and phenylsuccinyl-CoA transferase IaaL; Fig. 1). Because of the labile nature and fast hydrolytic degradation of the phenylsuccinyl-CoA regioisomers (Schühle et al. 2016), accumulation of these intermediates by a blocked degradation pathway would lead to the loss of the CoA-thioester activation. However, the known dual activities of IaaL as intramolecular phenylsuccinyl-CoA transferase and intermolecular CoA transferase between phenylsuccinyl-CoA and succinate would allow the cells to preserve both the activation energy of the thioester groups (via succinyl-CoA to ATP) and the degradation intermediate (in form of phenylsuccinate, resp. (2-aminophenyl)succinate), which can easily be re-activated using succinyl-CoA as CoA-donor when the metabolic block is overcome (Fig. 1). As indicated by data base searches and analyzing for related proteins, IaaF is a member of a special subclass of acyl-CoA dehydrogenases from bacterial and archaeal strains, which are all encoded in gene clusters coding for enzymes of indoleacetate metabolism (Schühle et al. 2016). A multiple alignment of these IaaF-related sequences with representative acyl-CoA dehydrogenases of various other subclasses revealed interesting variations of a highly conserved sequence motif close to their C-termini, which is known to be part of the active site in structurally characterized acyl-CoA dehydrogenases (Ghisla and Thorpe 2004). While most members of subclasses catalyzing oxidation of straight-chain alkyl-CoAs to α,β-unsaturated alkenoyl-CoA derivatives contain a conserved YEG motif, all members of the IaaF subclass deviate from this consensus and instead contain a GGG motif (Fig. 5).

**Fig. 6:**
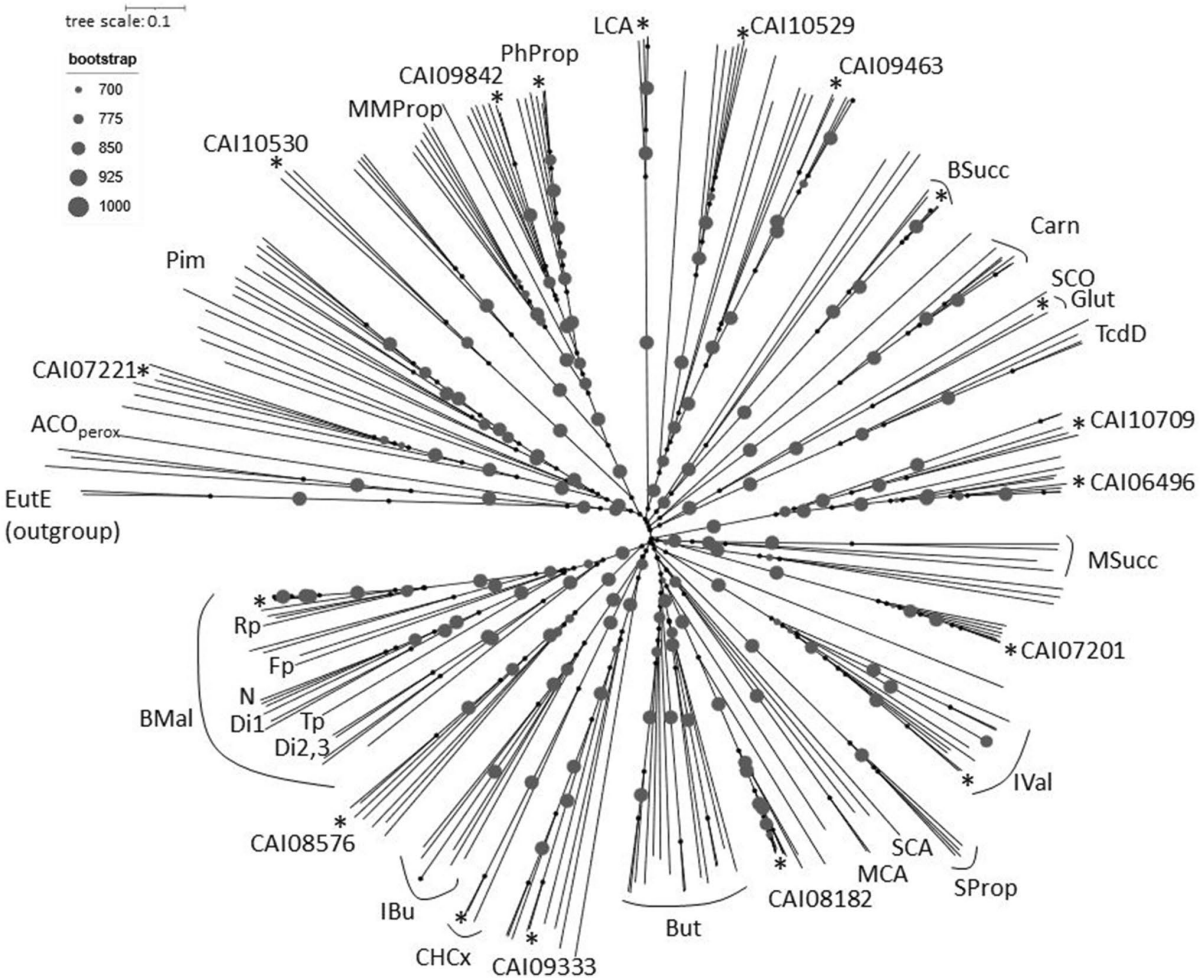
Similarity tree of acyl-CoA dehydrogenases and oxidases. Bootstrapping results ranging between 700 and 1000 (of 1000 repetitions) are shown as circles as indicated. Benzylmalonyl-CoA oxidizing IaaF orthologues have been labeled with BMal, and several representative source microbes are indicated as follows (with accession numbers): *, *A. aromaticum* (CAI07262); Rp, *Rhodopseudomonas palustris* (WP_011664634); Fp, *Ferroglobus placidus* (WP_012966786); N, strain NaphS2 (WP_006425528); Di1-3, three paralogues from *Desulfocarbo indianensis* (WP_049676703, WP_049676756, WP_049676580), Tp, *Thermoplasmatales* (EMR74870). Furthermore, all acyl-CoA dehydrogenases encoded in *A. aromatoleum* have been included and are indicated by asterisks (and accession numbers for those of unknown function). In analogy to BMal, all acyl-CoA dehydrogenase (DH) subfamilies with biochemically identified members have been labeled as follows (with representative accession numbers), whereas subfamilies consisting of enzymes of unknown function are unlabeled. ACO_perox_, peroxisomal acyl-CoA oxidase (1IS2); BSucc, benzylsuccinyl-CoA DH (CAI07171); But, butyryl-CoA DH (WP_010965998); Carn, butyrobetainyl-CoA DH (ANK05333); CHCx, cyclohexancarboxyl-CoA DH (CAI07205); Glut, glutaryl-CoA DH (CAI07810); IBu, isobutyl-CoA DH (1RX0); IVal, isovaleryl-CoA DH (CAI08760); LCA, long chain acyl-CoA DH (CAI07411); MCA, medium chain acyl-CoA DH (P11310); MMProp, methylmercaptopropionyl-CoA DH (Q5LLW7); MSucc, methylsuccinyl-CoA DH (CAX24659); PhProp, phenylpropionyl-CoA DH (CAI09150); Pim, pimelyl-CoA DH (CAI10529, CAI09347); SCO, short chain acyl-CoA oxidase (2IX5); SProp, sulfinopropionyl-CoA desulfinase (5AHS); TcdD, γ,δ-acyl-CoA DH (WP_006350839). EutE, propionaldehyde DH as outgroup (WP_011388669)

A similarity tree of IaaF orthologues with selected members from other acyl-CoA dehydrogenase subclasses shows that they occupy an isolated subbranch (labeled by BMal) separate from all other members of the family (Fig. 6). All enzymes of the BMal subbranch are encoded within operons containing further conserved genes of the *iaa* operon. Acyl-CoA dehydrogenases with other specificities as well as enzymes with unknown function occupy other subbranches of the family and can nicely be grouped based on their sequence conservation. Remarkably, most biochemically characterized enzymes retain either the active site YEG motif (But, MCA, Glut, SCO, MMProp in Fig. 6) or very similar motifs such as GEG (MSucc), GDG (CHCx), LEG (IBu), VEG (BSucc, LCA), IEG (TcdD) or FEG (ACO, TcdD). Substitution of the conserved Tyr, which is involved in binding the CoA-thioester substrates via main chain contacts, may help to accommodate α-substituted or otherwise bulky substrates like isobutyryl-CoA, isovaleryl-CoA, cyclohexanecarboxy-CoA, methyl- or benzylsuccinyl-CoA (Tiffany et al. 1997; Leuthner and Heider 2000; Battailem et al. 2004) in most of the latter enzymes. The conserved Glu of the YEG motif abstracts a proton from C-2 of the substrate and thereby initiates the reaction (Ghisla and Thorpe 2004). In addition to the IaaF orthologues, the Glu is missing in the enzymes of the subbranches IVal (GAG), SProp (AGG), Carn (SGG), and Pim (YGG), which can be explained by altered catalytic mechanisms in the former two cases, where structures are available. In isovaleryl-CoA dehydrogenases, another Glu takes over the function as proton-abstracting group (Tiffany et al. 1997) whereas sulfinopropionyl-CoA desulfinase attacks the sulfino group and eliminates it as sulfite in a completely different overall reaction (Schürmann et al. 2015). Remarkably, many of the still unassigned acyl-CoA dehydrogenases encoded in the *A. aromaticum* genome (CAI07201, 08576, 08182, 09333, 09396, 10529, 10530, 07221) also contain deviations from the YEG motif, possibly suggesting further unconventional roles of these enzymes.

**Fig. 7:**
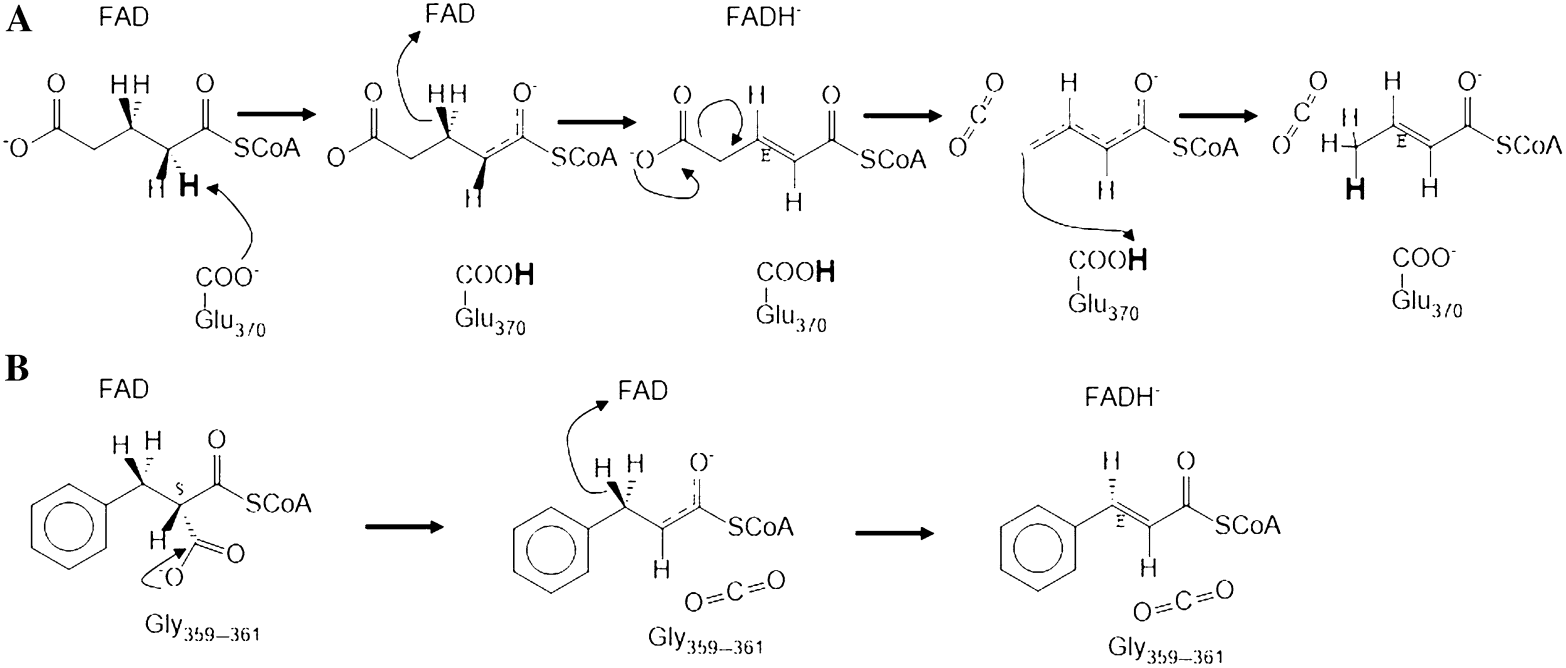
Proposed mechanisms of decarboxylating acyl-CoA dehydratases. **A** Two-step mechanism of oxidation and γ-carboxy group elimination by glutaryl-CoA dehydrogenase. **B** One-step process of simultaneous α-carboxy group elimination and oxidation by benzylmalonyl-CoA dehydrogenase. Note the proposed proton transfer from C2 to C4 of the substrate exerted by Glu_370_ of glutaryl-CoA dehydrogenase, which is replaced by Gly in benzylmalonyl-CoA dehydrogenase

The only other known decarboxylating acyl-CoA dehydrogenase in addition to IaaF is glutaryl-CoA dehydrogenase (Fu et al. 2004; Rao et al. 2006), which oxidizes glutaryl-CoA to crotonyl-CoA and CO_2_. This enzyme is well studied and contains the conserved YEG motif (Fig. 5). Its proposed mechanism proceeds in two sequential steps: first, the substrate is oxidized by deprotonation at C2 (via the conserved Glu_370_) and hydride abstraction from C3 via the FAD cofactor, then the transient enzyme-bound glutaconyl-CoA intermediate is decarboxylated with re-donation of the proton from Glu_370_ (Fig. 7A; Rao et al. 2006). In contrast, benzylmalonyl-CoA dehydrogenase does not contain the conserved YEG motif of the active site, which is replaced by GGG. We propose a mechanism of IaaF that does not involve abstraction of a proton from C-2, but achieves the same effect by directly eliminating the carboxy group from C-2 as CO_2_ (Fig. 7B). The missing Tyr side chain may allow for the necessary space to accommodate the α-carboxy group of benzylmalonyl-CoA, whereas the missing Glu side chain prevents unwanted deprotonation of C-2, while direct decarboxylation should be stimulated by the expected cavity formed by the three consecutive small and apolar Gly residues of the active site (Fig. 7B).
